# Towards a common code for difficulty: Navigating a narrow gap is like memorizing an extra digit

**DOI:** 10.3758/s13414-021-02356-4

**Published:** 2021-07-30

**Authors:** Iman Feghhi, John M. Franchak, David A. Rosenbaum

**Affiliations:** Department of Psychology, University of California, Riverside, CA USA

**Keywords:** Decision making, Mental effort, Metacognition, Physical effort, Task difficulty

## Abstract

What makes a task hard or easy? The question seems easy, but answering it has been hard. The only consensus has been that, all else being equal, easy tasks can be performed by more individuals than hard tasks, and easy tasks are usually preferred over hard tasks. Feghhi and Rosenbaum (*Journal of Experimental Psychology: Human Perception and Performance, 45*, 983–994, 2019) asked whether task difficulty might reflect a single amodal quantity. Based on their subjects’ two-alternative forced-choice data from tasks involving choices of tasks with graded physical and mental challenges, the authors showed that the difficulty of passing through a narrow gap rather than a wide gap was psychologically equivalent to memorizing an extra .55 digits. In the present study, we extended this approach by adding new arguments for the hypothesis that task difficulty might reflect a single amodal quantity (inspired by considerations of physics, economics, and the common code hypothesis for the study of perception and action), and we tested narrower gaps than before to see whether we would find a larger equivalent memory-digit. Consistent with our prediction, we obtained a value of .95. We suggest that our multi-modal two-alternative forced-choice procedure can pave the way toward a better understanding of task difficulty.

## Introduction

What makes a task hard or easy? Electrons have no trouble deciding. They take the path of least resistance, not knowing which way to go, but bunching up in areas of high resistance and veering toward areas of lower resistance. People behave similarly when heading for wide rather than narrow exits in theaters and stadiums. In both cases, the structure of the environment specifies path ease.

Physical systems are replete with such examples: Water tends to flow down steeper slopes, and light travels down least-time paths in accord with Fermat’s Principle (https://en.wikipedia.org/wiki/Fermat%27s_principle). Such examples illustrate a foundational principle of physics, the Law of Least Action (https://en.wikipedia.org/wiki/Principle_of_least_action).

The Law of Least Action has been applied to living systems, including human beings. In one of the best-known examples, the American linguist/mathematician George Kingsley Zipf (1949) offered the Law of Less Work. According to the Law of Less Work and as expressed here in our words, “The more common a word is, the shorter it is on average.” Consistent with the Law of Less Work, word frequency follows a power-function. The second-most common word is half as frequent as the most-common word, the third-most common word is half as frequent as the second-most-common word, and so on. If the Law of Less Work were not operative, information communication would be far less efficient than it is. As Zipf emphasized, communication, like light, minimizes time.

If the Law of Least Action holds for all the elements referred to above – electrons, water, light, and words – then it is natural to ask whether time, the fundamental value in all the cases listed, is sufficient to explain task difficulty? It might be, as several authors have suggested (Gray et al., 2006; Potts et al., 2018; Rosenbaum & Bui, 2019). Clearly, running at top speed for 10 min is harder than running at top speed for 5 min. However, a problem arises: Running at top speed for 10 min is also harder than walking for 11 min. Accordingly, time is dissociable from task difficulty (e.g., Kool et al., 2010).

Should the Law of Least Action be repealed, then, for human action? There may be a way to resolve the the problem associated with the walking-for-11-min-versus-running-for-10-min example. The time to rest and recover from an 11-min *walk* is less than the time to rest and recover from a 10-min *run*. Considering the full cycle time to engage and re-engage in the two tasks could explain why the 10-min run seems harder than the 11-min walk. If total time is considered, the short-duration run will seem harder than the long-duration walk, consistent with Fermat’s Principle and, by extension, the Law of Least Action.

Do people actually think about rest and recovery times when answering questions like this one? We can defer that question because time needn’t always be referred to in considerations of task difficulty. For example, time does not arise (in any obvious way) in connection with the shape of a chain suspended between two posts (a catenary). The arc form of the catenary reflects the Law of Least Action and is often used as an example of it. The shape of the catenary is the one that minimizes the difference between potential energy and kinetic energy (another way of expressing the Law of Least Action) and remains the same over time, provided there is no external disturbance.

These remarks suggest that, more likely than not, the Law of Least Action may underlie perceived task difficulty, even in view of evidence for various specific proposals about the currency underlying this psychological quantity, including energy (Craig, 2013), opportunity cost (Kurzban et al., 2013), and errors (Dunn et al., 2019). The central claim of this paper is that it may be pointless to try to distinguish among particular alternative accounts of task difficulty even though thinkers from many disciplines have tried to do so, including people working in philosophy, sport science, psychology, language, education, and robotics (André et al., 2019; Burgess & Jones, 1997; Cos, 2017; Fisher & Steele, 2014; Halperin & Emanuel, 2020; Montero, 2016; Pageaux, 2014; Shenhav et al., 2017; Song et al., 2019; Steele, 2020). We think it is very unlikely that one account will be correct in all circumstances because context always matters. For example, people may be willing to pay a lot of money for the most relaxing ride possible (one end of the energy continuum) or for membership in a gym affording the most intense workout imaginable (the opposite end of the continuum). Notwithstanding such circumstantial changes, we hypothesize that within a bounded set of circumstances, a single quantity might be able to explain task ease. We are especially interested in the possibility that the quantity might be abstract and amodal. In much the same way that the difference between potential energy and kinetic energy – an abstract quantity and not one that can be directly or immediately sensed – appears to underlie all of physical efficiency, the true measure of task difficulty might be similarly abstract. We aim here to test for such a quantity. Our pursuit is motivated not just by physics and related fields, but also by economics, where value is treated as an abstract quantity (https://en.wikipedia.org/wiki/Theory_of_value_(economics)), and, closer to home, the demonstration of a common code for perception and action (Prinz, 1990; Prinz & Hommel, 2002). The common code hypothesis for perception and action has inspired us to hypothesize that there is, likewise, a common code for difficulty.

## Lead-up to the present two experiments

In this article, we will report two experiments based on an earlier pair of experiments by Feghhi and Rosenbaum (2019). These authors inquired into the possibility that task difficulty might reflect a single abstract quantity. They provided university students with two task options, each of which had varying degrees of physical and mental demands. The participants chose between carrying an empty box through a wide gap (81 cm) or a narrow gap (36 cm), having memorized six, seven, or eight digits before passing through either gap. The instruction was to do whatever seemed easier – memorizing the list associated with the wide gap and then going through that gap, or memorizing the list associated with the narrow gap and then going through that gap, knowing that the list that had been memorized would have to be recalled upon reaching the other side. Each list length was offered with each gap size in all possible pairs and with the wide or narrow gap on the right or left for all participants. From the obtained two-alternative forced-choice data, Feghhi and Rosenbaum estimated the point of subjective equality for the wide and narrow gap, expressed in number of digits. They found that going through the narrow gap was functionally equivalent to memorizing an extra .55 digits.

In their second experiment, Feghhi and Rosenbaum (2019) tested a fresh sample of participants on the same tasks, except that now they dictated to those subjects which task should be done. In that case, the obtained performance data (i.e., the error rates) were virtually identical to what they were in the choice condition. This outcome provided assurance that the results of the first experiment were not biased by unequal numbers of observations in the conditions for which data existed. Beyond that and more importantly, Feghhi and Rosenbaum concluded that having to choose a task or having been told what to do did not affect accuracy; subjects had made wise choices when they could choose.

The two experiments reported here were modeled on the two that Feghhi and Rosenbaum (2019) conducted.^1^ In the present Experiment 1, subjects chose walking paths and associated memory loads, as in the earlier study. In the present Experiment 2, subjects were assigned each of the walking paths and associated memory loads of the first experiment, as in the 2019 study. The new feature of the present experiments was that we used a narrower gap than the narrower gap used before. We were motivated to do so because navigation errors (bumping into an obstacle while passing through the narrow gap) were rare in the 2019 experiments.^2^ We used a narrower gap here to challenge the perceptual-motor system more than in Feghhi and Rosenbaum’s (2019) study. We predicted that by making the narrow gap narrower we would increase the navigation error rate and, more importantly, would elevate the point of subjective equality for the wide and narrow gap, expressed in number of digits. Whereas Feghhi and Rosenbaum found that going through the narrow gap was functionally equivalent to memorizing an extra .55 digits, we predicted that with an even narrower gap, this value would increase. By how much we could not say; too little data exist in this line of work to allow for a more precise prediction.

A further refinement of the method was that we tailored the gap sizes to individual subjects. To do so, we took advantage of the apparatus and expertise of Franchak (2017, 2020) and Labinger et al. (2018), who studied gap clearance using a sophisticated apparatus that had two gaps with sliding doors. This apparatus allowed the widths of the openings to be adjusted with high resolution (0.5-cm increments) to befit, or not, the features of individual subjects. As in the previous work with this apparatus, we were interested in adjusting the width of the aperture to each individual’s body size.

## Experiment 1

### Method

#### Participants

Forty-two undergraduate students (32 female and 10 male) from the University of California, Riverside (UCR), participated in this experiment for course credit. The participants ranged in age from 18 years to 24 years, with an average of 19.41 years and a standard deviation of 1.04 years. All participants signed an informed consent form before the experiment. The current sample size was similar to the sample size of Feghhi and Rosenbaum (2019), who tested 40 subjects. That number let us exceed the value of *n* = 500 observations recommended for evaluation of logistic regression models (Cohen, Cohen, West, & Aiken, 2013; Hosmer, Hosmer, Le Cessie, & Lemeshow, 1997). With 40 subjects, the number of choices per participant was 18, so there were 720 observations altogether. Two more subjects offered their services here via the UCR psychology subject pool (where students get course credit for participating). We were happy to have 42 subjects rather than 40 subjects in this experiment.

#### Apparatus

**Fig. 1:**
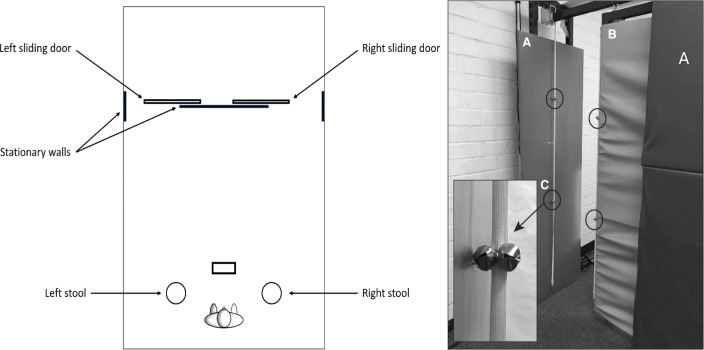
Setup in Experiment 1. The left panel shows a schematic birds-eye view of the apparatus. The right panel shows the left doorway. In the left panel, the left doorway is narrow and the right doorway is wide. There are three stationary walls, one in the middle and one on each side. The right panel shows the stationary wall (**a**) parallel to the sliding wall (**b**) as well as the other stationary wall (**c**). The magnified inset in the right panel shows one of the four pairs of bells located at the circled areas

As shown in Fig. 1, at the start of each trial, participants stood at a home position and saw two lists of digits. One list lay on a stool 90 cm to the left and another list of digits lay on a 90 cm stool to the right. An empty box (48 × 48 × 10 cm) stood on a platform (a music stand tilted to be parallel to the ground, 95 cm high above the floor) in front of the subject, who could see two doorways 275 cm away from the home position. The widths of the doorways could be adjusted between 0 and 70 cm (with a resolution of .5 cm) by sliding the doors (185 cm tall × 100 cm wide) along a perpendicular stationary wall (182 cm tall × 62 cm wide). A locking mechanism prevented the doorways from moving when the mechanism was engaged.

On each trial, one of the doorways was kept at a fixed width of 70 cm, which was the widest possible width. We assumed that navigating through the wide doorway provided minimal challenges for all the participants. The other doorway’s width was adjusted based on each participant’s body size. The narrow doorway’s width was based on a calibration process such that each participant had a 50% chance of bumping the edges of the doorway. To detect bumping, two elastic bands were aligned with the edges of the doorways. Small bells were attached to the bands and participants were told to avoid bumping into the elastic bands to prevent the bells from ringing.

After passing through either doorway, the participant attempted to recall the digit list in the presence of an experimenter who awaited the participant’s arrival. The experimenter stood between the two doorways and was unable to see the participant until s/he entered the post-doorway area. After the recall phase, the experimenter opened the doorway all the way, so on the way back, the participant did not have to pass through a narrow doorway.

#### Procedure and design

After signing the consent form, each participant went through the calibration process that was used to determine the doorway width for each participant that was narrow enough for each of them to have a 50% chance of making a mistake in passing through it (i.e., bumping into an elastic band, causing a bell to ring). Clearly, the participants’ body sizes were only one factor in determining the narrow doorway width. Spatial awareness, controlling body sway, dynamic balance, and practice were also determinative factors. We did not attempt to determine which of these factors contributed to any participant’s doorway width.

A single doorway was used during the calibration trials. On each calibration trial, it was set to a width between 35 cm and 60 cm in 0.5-cm increments that participants were requested to attempt to pass through. They were instructed to turn their body and walk sideways to clear the doorway. The doorway width on each trial was set to find the 50% threshold based on the outcome of the previous trial (successfully passing through vs. bumping into the side of the doorway). Over the first five trials, a binary search procedure was used, as in Franchak et al. (2010), to find a doorway width close to the 50% point. Another 15 trials were then used to further adjust the doorway width, decreasing it by 1.5 cm or increasing it by 2 cm if the participant succeeded or failed, respectively, on the previous trial. A cumulative Gaussian function was fitted to the data from the calibration trials using the Palamedes Toolbox (Prins & Kingdom, 2018). Ultimately, the doorway width that was used as the narrow width in the main experiment per participant was the width for which that participant could pass through the doorway without causing a bell to sound 50% of the time.

After the calibration process, the participant was asked to stand at the home position. There they saw digit lists (six-, seven-, or eight-digit random numbers), each of which was printed on a piece of paper and placed on a stool to the subject’s left and right. A box (empty rectangle in the left panel of Fig. 1) stood on another stool (95 cm height) directly in front of the subject and within easy reach. The box was empty and measured 48 × 48 × 10 cm. The subject could also see the two doorways (275 cm away), one to the right and one to the left. One of the doorways was always wide (70 cm) and the other was always narrow, set individually for the subject based on the calibration procedure described above. For a random half of the participants, the right doorway was wide for the first nine trials and narrow for the next nine trials. For the other participants, it was the other way around. All participants had 18 trials for the nine conditions. As a result, the choice data per condition had two observations per participant. With 42 participants, this meant that the possible proportions per condition (i.e., the possible values of *p*(Wide), the probability of choosing the wide gap) were 0/84, 1/84, …, 84/84. The information content was therefore log_2_(85) = 6.40 bits.

The participants’ task was to do whatever seemed easier, memorize the digit list on the left and then carry the box through the left door, or memorize the digit list on the right and then carry the box through the right door. Participants were told that there was no time limit for memorizing the digit lists or for passing through the door and setting the box down on the target platform. Once they thought they had memorized the lists, they picked up the box and started walking toward the selected doorway. After passing through the chosen door and setting the box down, they tried to recall the digits of the list for the side they had chosen, having been told that order mattered; the digits were to be recalled in the left-right order in which they appeared. Participants were told that if they made a mistake, they would have to redo the trial. A mistake was defined as causing a bell to ring while passing through a door or misrecalling the digits in any way (i.e., naming a digit not on the list or recalling the digits in the wrong order). If a mistake was made in a redo trial, the trial did not have to be repeated again.

### Results

#### Number of choices and error rates

Table 1 shows the number of times the tasks with different door widths and memory loads were chosen as well as the associated error rates. As seen in Table 1, the wide-door option was chosen more often than the narrow-door option, and paths with smaller memory loads were chosen more often than paths with larger memory loads. In addition, error rates of any kind were inversely related to the number of chosen options.

**Table 1:** Main results of Experiments 1 and 2 in the six conditions

	Experiment 1				Experiment 2			
Condition	N	*p*(Error)	*p*(R)	*p*(N)	N	*p*(Error)	*p*(R)	*p*(N)
Wide-6	236	.11	.11	0	84	.13	.13	0
Wide-7	190	.25	.25	0	84	.19	.19	0
Wide-8	120	.33	.33	0	84	.35	.35	0
Narrow-6	129	.40	.20	.29	84	.38	.22	.31
Narrow-7	73	.47	.30	.29	84	.48	.28	.29
Narrow-8	44	.61	.41	.32	84	.57	.39	.33

The entries are the number of trials, N, in which each door width and memory load combination was chosen; the probability, *p*(Error), of an error of any kind; the probability, *p*(R), of a recall error; and the probability, *p*(N), of a navigation error

Regarding the two kinds of errors, the probability of recall error, *p*(R), was inversely related to the number of chosen options. No navigation errors occurred when participants passed through the wide doorway. When participants passed through the narrow doorway, the memory load had little or no effect on the probability of a navigation error, *p*(N).

To analyze the effect of physical and mental demands on error rate, we conducted a General Estimating Equations (GEE) analysis of the probability of any kind of error, *p*(Error), and the probability of error in recall, *p*(R). We did not conduct a GEE analysis on the probability of error in navigation, *p*(N), because *p*(N) in the wide gap was 0. When a predictive variable perfectly predicts the outcome (in our case, going through the wide gap perfectly), there is a “quasi-complete separation” problem (Albert & Anderson, 1984), which makes the maximum likelihood calculation impossible. With the GEE analysis, using a 2 (wide and narrow doorways) × 3 (six, seven, and eight digits) design, we found that *p*(Error) showed a main effect of memory load, Wald chi-square = 4.32, *p* = .03, such that *p*(Error) with memory load of six (.22 95% confidence interval (CI) [.17 .28]) was lower than *p*(Error) with memory load of seven (.35, 95% CI [.27, .44]) and was lower than p(Error) with memory load of eight (.47, 95% CI [.37, .58]). There was a main effect of doorway width, Wald chi-square = 6.38, *p* = .01 such that p(Error) for the wide doorway (.21, 95% CI [.16, .28]) was lower than p(Error) for the narrow doorway (.49, 95% CI [.40, .59]). There was no interaction between door width and memory load, Wald chi-square = 2.33, *p* = .12. A 2 (wide and narrow doorways) × 3 (six, seven, and eight digits) GEE analysis on *p*(R) showed a main effect of memory load, Wald chi-square = 6.83, *p* = .009, no main effect of doorway width, Wald chi-square = 1.78, *p* = .18, and no interaction between these factors, Wald chi-square = 0.96, *p* = .32.

#### Choices

Whereas Table 1 showed the total number of times that participants chose a task option with the characteristics listed per row, those numbers do not break down how often each task option was chosen depending on the other task with which it was paired. Table 2 shows the relevant data, now expressed in proportions rather than total numbers. The table shows the probability, *p*(Wide), of choosing the wide doorway depending on the number of digits to be memorized for the wide versus narrow doorway.

**Table 2:** Probability of choosing the wide gap, *p*(Wide), in the nine memory load conditions of Experiment 1 (along with 95% confidence intervals)

Wide gap	Narrow gap		
	6	7	8
6	.82 (.74, .90)	.94 (.89, .99)	.92 (.86, .98)
7	.49 (.38, .60)	.77 (.68, .86)	.90 (.83, .96)
8	.23 (.14, .32)	.45 (.35, .56)	.68 (.58, .78)

As seen in Table 2, the wide gap was chosen less often as its associated memory load increased. The values decreased from the first row down to the third. The wide gap was chosen more as the narrow gap memory load increased. The values increased from the first column to the last.

We sought to put these values together into a single mathematical model whose constructs could be related to the putative steps involved in choosing the task alternatives. We assumed that, by default, participants preferred the wide door, but if the difference between the wide-door memory load and the narrow-door memory load exceeded a threshold, the preference would switch to the narrow door, doing so with increasing probability the greater the difference between the narrow-door memory load and its threshold. To express the model in an equation, we used a logistic function with two free parameters, the critical memory-load difference or switching point, *S*, and the decisiveness of the decision, visualized as the steepness, *K*, of the curve:

$$ p(Wide)=\frac{1}{1+{e}^{-K\left(x-S\right)}} $$

**Fig. 2:**
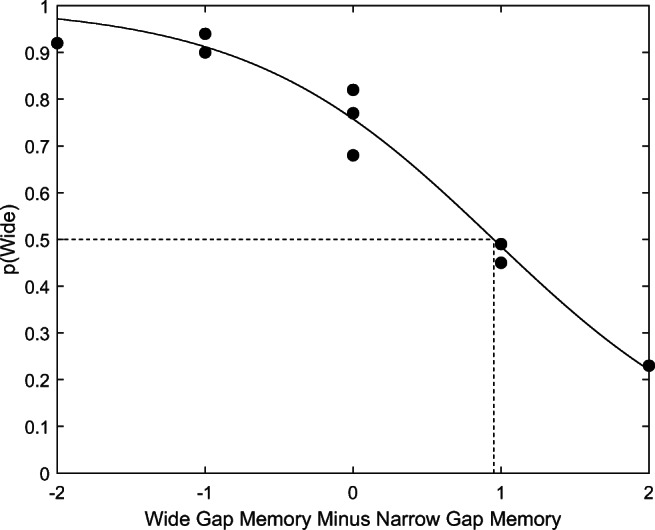
Probability of choosing the wide gap, *p*(Wide), as a function of the difference between the memory load of the two doorways. The black dots show the observed probabilities (aggregated single values of 0 or 1 for each participant), and the curve shows the model’s best fit. The dashed lines show the switch point. Multiple black dots appear at some horizontal positions because there were multiple conditions with that memory load difference. There were two such conditions for the differences of -1 and 1, and three such conditions for the difference of 0. There was only one condition for which the memory was -2, and only one condition for which the memory was +2

The best fit is shown in Fig. 2. The parameter values that provided the best fit were *S* = 0.95 and *K* = 1.2. The interpretation of *S* = .95 was that going through the narrow doorway was equivalent, in terms of difficulty, to memorizing an extra .95 digits on average. The model accounted for *R*^*2*^ = .97 of the variance in the observed probabilities.

## Discussion

The purpose of Experiment 1 was to replicate the first experiment of Feghhi and Rosenbaum (2019) using a narrower gap than the narrow gap of the 2019 study. Although the original narrow gap yielded a few navigation errors in the 2019 report – subjects bumped into the edge of the narrow gap at most 5% of the time – we predicted that a more challenging navigation task would give rise to more navigation errors and, more interestingly, a rise in the estimated memory-load equivalence. Using an adaptive procedure to set the width of the narrow aperture, we succeeded in increasing the likelihood of navigation errors, though we failed to get the navigation errors up to *p*(N) = .5. Possibly, practice navigating through the gap during the calibration task helped participants improve subsequent navigation. But more importantly and more interestingly, we obtained a rise in the associated memory-load estimate, from .55 in the 2019 study to .95 in the present study. By considering the unexplained variance of the logistic function when S was set to .95 (the best value in this study) versus .55 (the best value in the 2019 study), and keeping K at 1.2 in both cases, we determined that the present data were 4.97 times more likely to have come from a logistic function whose *S* value was .95 than from a logistic function whose *S* value was .55. The method we used to arrive at this value was the one introduced by Glover and Dixon (2004).

## Experiment 2

The second experiment was designed to address the same question as the one addressed in the second experiment of Feghhi and Rosenbaum (2019): Did participants’ choices reflect their actual abilities? A subordinate, less interesting, question was whether the choice data were unduly influenced by unequal numbers of observations in the choices made? It was possible that they could have been.

As in the earlier study, we eliminated choices in Experiment 2 and asked participants to do each of the possible tasks that were available to the participants in Experiment 1. We reasoned that if participants’ choices reflected their actual abilities and if the choice data were not unduly influenced by unequal numbers of observations in the choices provided, the error data of Experiment 2 would be the same as the error data of Experiment 1.

### Method

#### Participants

Forty-four undergraduate students (33 female and 11 male) from the University of California, Riverside, participated in this experiment for course credit. The participants ranged in age from 18 years to 24 years, with an average age of 19.98 years and a standard deviation of 1.29 years. All participants signed the informed consent form before the experiment. The larger number of subjects in this experiment compared to the last one was simply motivated by wanting to help students get their needed academic credit for their Intro-Psych class. As before, we were happy to test a few more subjects who volunteered than were strictly required or invited.

#### Apparatus

The apparatus was the same as Experiment 1, but, at the start of each trial, only one of the stools had a set of six, seven, or eight random digits on it. The side of the stool indicated the doorway to be traversed.

#### Procedure and design

At the start of each trial, the experimenter put a piece of paper on the left or right stool. Participants were asked to memorize the digit list, pick and carry the empty box through the doorway on the corresponding side, and then recall the numbers. For a random half of participants, the left doorway was narrow in the first trials and wide in the last trials. For the other half of the participants, the order was reversed. The same calibration procedure for determining the door width per participant was used here as well.

### Results and discussion

#### Error rates

**Fig. 3:**
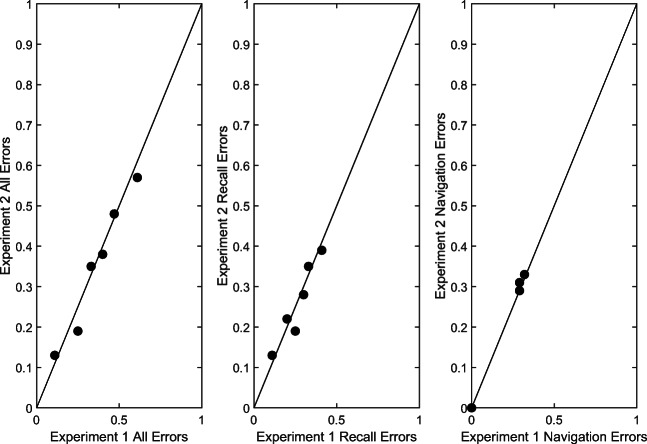
Error rates in Experiment 2 plotted as a function of error rates in Experiment 1. The solid diagonal lines are the identity lines. The leftmost graph is for all errors, the middle graph is for recall errors, and the right graph is for navigation errors

The data from this experiment have already been shown in Table 1. As seen there, the error rates in Experiment 2 were remarkably similar to the error rates in Experiment 1. This is shown in graphical form in Fig. 3.

To test the similarity between the two sets of results, we conducted a 2 (wide and narrow doorways) × 3 (six, seven, and eight digits) × 2 (experiments) GEE analysis on *p*(Error). The results showed a main effect of memory load, Wald chi-square = 44.82, *p* < .001, a main effect of doorway width, Wald chi-square = 71.082, *p* = .01, but no effect of experiment Wald chi-square = 0.12, *p* = .73.

The result was clear. Removing the choices, which forced performance of the indicated tasks an equal number of times per condition, yielded the same pattern of errors in the two experiments. Participants’ choices in Experiment 1 reflected their actual abilities.

## General discussion

In this article, we described two experiments aimed at establishing the relation between two different kinds of variables: the difficulty of a perceptual-motor task, and the difficulty of a mental (memory) task. We reasoned that if these two kinds of variables could not be compared, people would be unable to choose between them in a systematic fashion; their choices would be chaotic, and scientists like us would be unable to make principled predictions about the choice data we obtain. Our results let us reject this hypothesis. We made a specific prediction and we obtained data consistent with it.

We asked participants to choose and perform the easier of two options: memorizing six, seven, or eight random digits and going through a wide gap, *or* memorizing six, seven, or eight random digits and going through a narrow gap. In an earlier study, Feghhi and Rosenbaum (2019) introduced this task with gaps that were 81 cm wide and 36 cm wide. Feghhi and Rosenbaum found that participants were willing, on average, to memorize .55 more digits to avoid the narrow gap. In the present experiment, we made the narrow gap narrower and found that participants were willing, on average, to memorize .95 more digits to avoid the narrow gap. We reached this estimate by fitting a logistic function to the choice data. According to the process model underlying the logistic function, participants would prefer the wide gap by default but would switch to the narrow gap if the wide-gap memory load exceeded a threshold value. That value turned out to be .95 digits. We could show that our choice data were nearly five times more likely to have come from a source in which participants were willing to pass through the narrow gap when its memory load had .95 fewer items than when its memory load had .55 fewer items, which was the estimate from the previous experiment. Similarly, we could show – and this is a new statistic, not reported earlier in this article – that the choice data from the previous experiment were 8.42 times more likely to have come from a source in which participants were willing to pass through the narrow gap when its memory load had .55 fewer items than when its memory load had .95 fewer items.

In the remainder of this *General discussion*, we take up five remaining issues: (1) the relation between *p*(Wide) and *p*(Error); (2) the possibility that mappings between memorial difficulty and physical difficulty may suffice without positing an abstract, amodal representation of difficulty per se; (3) the value of pursuing numerical values in research about action, perception, and psychophysics as well as related fields; (4) the promise of our approach, with special reference to the use of the two-alternative forced-choice procedure; and (5) the limitations of the present study.

Regarding the first issue, the relation between *p*(Wide) and *p*(Error), it is interesting to pursue the possibility that in Experiment 1, these two variables had a simple relation and, moreover, that when *p*(Wide) was plotted as a function of *p*(Error), the point of subjective equality would land squarely on *p*(Error)=.5. Such an outcome would accord with the hypothesis that the decision to go through the wide or narrow gap was based on the desire to minimize error, for at *p*(Error)=.5 the likelihood of error would be indistinguishable for the two gaps. It is certainly plausible that the desire to minimize error could be the sole driver of choice. Dunn et al. (2019) proposed that the more error-prone a task, the more difficult it is perceived to be. It is also known that similar brain regions are active after making a mistake (Baker & Holroyd, 2011; Miltner et al., 2003) and in value evaluation and effort exertion (Apps et al., 2015; Apps & Ramnani, 2014; Mulert et al., 2005; Shenhav et al., 2013; Walton et al., 2003). These observations indicate that errors can be perceived as costly and therefore to be avoided.

When we fitted a logistic function to data points for *p*(Wide) plotted as a function of *p*(Error), we found that the coefficient of determination, *R*^*2*^, was comparable to what it was for the logistic fit in Fig. 2 (very high). However, we found that the point of subjective equality was at *p*(Error)=.39 rather than at *p*(Error) = .50. This result is not consistent with the hypothesis that the choice of gap was solely designed to reduce *p*(Error). Interestingly, analogous results were also found by Feghhi and Rosenbaum (2019) and by Feghhi and Rosenbaum (2020), who placed so much weight on this finding that they entitled their article “Effort avoidance is not simply error avoidance.” This conclusion makes sense considering that not all errors are equally costly. Slipping off a stone in one’s garden has a very different cost to slipping off a ledge on the edge of a cliff with a thousand-foot chasm beneath it.

Regarding the second issue, the possibility that mappings between memorial difficulty and physical difficulty may suffice without positing an abstract, amodal representation of difficulty per se, we cannot rule out this possibility for the data we have. Conceivably there may be values of memorial difficulty and values for physical difficulty with some mathematically well-defined mapping between the two, with no intervening representations. On the other hand, neural network modeling has shown that neural networks capable of reasonably complex learning must have an intermediate hidden layer as well as an input layer and an output layer. In our case, the input layer could be for memory difficulty and the output layer could be for physical difficulty; we have no way of distinguishing between these possibilities and have no reason to try. The important point is that the intermediate hidden layer would be task difficulty. Given that intermediate hidden layers are well known to be essential for successful neural modeling of attention, perception, and psychophysics, it is hardly surprising that extensive evidence exists for an abstract, amodal common code for perception and action (Prinz, 1990; Prinz & Hommel, 2002). We therefore think that difficulty is also represented in some abstract, amodal common code probably represented in a hidden intermediate layer of the relevant neural substrate.

Regarding the third issue, the value of pursuing numerical values for research in this area, we have been moved by recent arguments from Yarkoni and Westfall (2017), who have suggested that models in psychological science should be able to predict new numerical values, much as physics and other sciences have long done. The numerical prediction we made here was primitive by the standards of physics, for all we could predict was that the value of S would be larger than in the predecessor study. That prediction was supported, suggesting we were on the right track. In a future study, we might next ask a more subtle question such as this: Over a range of narrow gap sizes in a within-subject design, with six, seven, or eight memory items per choice and a fixed-width wider gap, how will S vary with the size of the narrow gap? Will S be a linear function of the narrow gap size or a logarithmic function? Science progresses by answering questions of this sort.

Regarding the fourth issue, concerning the promise of our approach with special reference to the use of the two-alternative forced-choice procedure, we would like to point out that the procedure we have used here has proven, time and again, to yield lovely, interpretable data (e.g., Rosenbaum et al., 2013). We have sufficient faith in the two-alternative forced-choice procedure to recommend it to others interested in assessing the perception of task difficulty, both in basic research, where it can add to the understanding of multi-modal experience and in applied contexts, where it can be useful. For example, in clinical settings, the method can be used to show how patients view the difficulty of performing a task. If hemiparetic patients judge the difficulty of moving an affected arm as being comparable to the difficulty of memorizing *five* digits soon after stroke but as being comparable to memorizing *two* digits later on, that outcome can provide a quantitative index of the change in the judged difficulty of the arm-movement task. If it is clear that the memory abilities remain the same, the measured change can be used to gauge recovery and design future treatments.

We turn finally to the fifth issue, the limitations of the present study. In the current work, we investigated a small range of memorization demands (six, seven, and eight digits) and only two levels of navigation demands. Based on the common code hypothesis, these two demands should be lawfully comparable in other ranges as well. That said, we make no claim how the relationship would change outside the ranges used here – for example, whether the perceived difficulty of the same navigation challenge would be similar to what we measured here if we used two-, three-, and four-digit lists. This topic needs more investigation.

The common code hypothesis also predicts that other aspects of a task, like energy expenditure, time, utility, and consequence of mistakes, should be convertible to the perceived difficulty and hence be systematically comparable. Given that different demands have different levels of evaluability (Dunn et al., 2017), further experiments are needed to better understand how different demands are compared. We did not explore all of these potential contributors to perceived difficulty. For example, we did not track possible differences in speed-accuracy tradeoffs.

Lastly, measuring each participant’s digit span could help reveal the impact of navigation on memory performance. Measuring each participant’s digit span could also be used to equate the memorization challenge across participants and thereby have more control over the demands of the memorization tasks. Pursuing this question, like the others raised above, should help advance understanding in this area of study.

## Data Availability

The datasets generated during and/or analyzed during the current study are available from the corresponding author on reasonable request.

## Footnotes

1 Another pair of experiments, by Feghhi and Rosenbaum (2020), replicated the main features of the 2019 results, but aspects of the 2020 procedure were sufficiently different from those of the 2019 report (using auditory inputs rather than visual inputs for the to-be-memorized materials) that we merely mention the replication here in passing.

2 There were no navigation errors at all while passing through the wide gap in the earlier experiments, and this fact let us use a slightly narrower wide gap here, owing to the pre-existing physical structure of the apparatus for the present experiments. Whereas the wide gap was 81 cm wide before, here it was 70 cm. We expected no navigation errors with the slightly narrower wide gap here.
